# Expression of Staphylococcal Virulence Genes In Situ in Human Skin and Soft Tissue Infections

**DOI:** 10.3390/antibiotics11040527

**Published:** 2022-04-14

**Authors:** Michael S. Pulia, Jennifer Anderson, Zhan Ye, Noha S. Elsayed, Thao Le, Jacob Patitucci, Krishna Ganta, Matthew Hall, Vineet K. Singh, Sanjay K. Shukla

**Affiliations:** 1 Department of Emergency Medicine, University of Wisconsin, School of Medicine and Public Health, Madison, WI 53726, USA; mspulia@medicine.wisc.edu; 2 Integrated Research Development Center, Marshfield Clinic Research Institute, Marshfield, WI 54449, USA; anderson.jennifer@mcri.mfldclin.edu (J.A.); le.thao@mcrf.mfldclin.edu (T.L.); 3 Bioinformatics Research Center, Marshfield Clinic Research Institute, Marshfield, WI 54449, USA; yzharold@gmail.com (Z.Y.); jacobpatitucci@gmail.com (J.P.); 4 Center for Precision Medicine Research, Marshfield Clinic Research Institute, Marshfield, WI 54449, USA; elgendy.noha@marshfieldresearch.org (N.S.E.); ganta.krishna@mcrf.mfldclin.edu (K.G.); 5 Department of Infectious Diseases, Marshfield Clinic Health System, Marshfield, WI 54449, USA; hall.matthew@marshfieldclinic.org; 6 Department of Microbiology and Immunology, Kirksville College of Osteopathic Medicine, A.T. Still, University of Health Sciences, Kirksville, MO 63501, USA; vsingh@atsu.edu

**Keywords:** *Staphylococcus aureus*, SSTI, Panton–Valentine leukocidin, hemolysins, clumping factors, accessory gene regulators

## Abstract

Background: *Staphylococcus aureus*, the most common pathogen in skin and soft tissue infections (SSTI), harbors many well-characterized virulence genes. However, the expression of many of them in SSTIs is unknown. In this study, *S. aureus* virulence genes expressed in SSTI were investigated. Methods: Fifty-three subjects presenting to the outpatient’s care and emergency departments with a purulent SSTI at two medical centers in Wisconsin, USA, were enrolled in the study. Total mRNA was extracted from the purulent or swab materials, made into cDNA and sequenced on MiSeq platform. The relative cDNA counts to *gmk* and identifications of the transcripts were carried out with respect to USA300 reference genome and using SAMTOOLS v.1.3 and BWA, respectively. Result: A significantly higher cDNA count was observed for many of the virulence and regulatory gene transcripts in the pus samples compared to the swab samples relative to the cDNA counts for *gmk*, a housekeeping gene. They were for *luk*S-*PV* (18.6 vs. 14.2), *isa*A (13.4 vs. 8.5), *ssa*A (4.8 vs. 3.1), *hlg*C (1.4 vs. 1.33), *atl* (17.7 vs. 8.33), *clfA* (3.9 vs. 0.83), *eno* (6.04 vs. 3.16), *fnbA* (5.93 vs. 0.33), *saeS* (6.3 vs. 1.33), *saeR* (5.4 vs. 3.33) and *agrC* (5.6 vs. 1.5). Conclusions: A relative increase in the transcripts of several toxins, adhesion and regulatory genes with respect to a *gmk* in purulent materials suggests their role in situ during SSTIs, perhaps in an orchestrated manner.

## 1. Introduction

*Staphylococcus aureus*, a Gram-positive bacterium is a major human commensal in the anterior nares and other moist body sites and can be present in approximately 30% of the human population [1,2]. *S. aureus* commensalism is a major risk factor for future clinical infections when protective barriers of the skin and innate immunity are breached and it is able to penetrate deeper into body tissues. In addition to skin and soft tissue infections (SSTI), it is also capable of causing a variety of other invasive diseases such as pneumonia, osteomyelitis, and bacteremia [3,4,5,6]. Treatment of these infections is a challenge as clinical *S. aureus* isolates express resistance to most of the anti-staphylococcal antibiotics, including the *β*-*lactam* class [7]. *S. aureus* persists within the host due to its ability to produce a large number of virulence factors and biofilm that enables this bacterium to establish an infection, extract nutrients from the host, and evade immune recognition and clearance [8]. Additionally, recurrence and persistence of *S. aureus* SSTIs is a common clinical phenomenon and an area of major concern [9].

In the past three decades, there has been continued increase in the incidences of community-acquired methicillin-resistant *S. aureus* (MRSA) infections [10] and clonally, the majority of these MRSA SSTIs have been due to *S. aureus* strain, USA300 or USA300 lineage strains [11,12]. Although, these strains harbor genes for several well-described (e.g., Panton–Valentine leukocidin and α-toxin) and other putative virulence factors such as *Escherichia coli* ampicillin resistance (*ear*) [13], lipoprotein lipase (*lpl*10) [14] and some of the staphylococcal superantigen-like proteins [15,16], it is unclear which of these are expressed in the SSTI wounds. To determine the distinctive virulence potential of clinical and carriage *S. aureus* isolates, including MRSA, many of the earlier studies simply profiled the known and putative virulence genes by annotating the genome or screening specific genes by PCR. For example, besides annotating known virulence genes, Baba et al. also identified 19 putative virulence genes in the CA-MRSA strain, MW2 based on their signature sequence motifs [14]. Similarly, other studies also described the virulence genes profile of *S. aureus* isolated from carriage and clinical sources to correlate the bacterial genotype with its clinical phenotype [17,18,19,20,21,22,23]. Since the presence and absence of virulence genes in an isolate do not necessarily confirm its expression and true virulence potential, some studies investigated the expression of specific virulence genes using in vitro culture, which again may not correlate to the in vivo conditions. Said-Salim et al. investigated the expression of PVL genes in vitro from several CA-MRSA isolates and noted variability in its expression levels in different strains [24]. The present study aimed to detect the transcript levels of putative toxin genes and virulence genes directly from purulent material following surgical drainage or from wound material using a swab.

The goal of this exploratory study was to identify the known and putative virulence genes that are expressed in situ in *S. aureus* present in SSTI. The study utilized purulent material or wound swabs collected from acute SSTIs, which were analyzed using a transcriptomic approach to measure gene expression through high-throughput sequencing. In this preliminary characterization, we show several *S. aureus* genes expressed during SSTI thereby implicating their potential role in SSTI pathogenesis.

## 2. Materials and Methods

### 2.1. Subjects and Research Specimens

Patients presenting with a purulent SSTI requiring surgical drainage were enrolled during outpatient visits to the Marshfield Clinic or the emergency department of the University of Wisconsin–Madison. Informed consent was obtained from all participants enrolled in the study. Using a sterile needle and syringe, a pus sample was collected if the SSTI had larger wounds and there was sufficient volume of purulent material. In cases where there was an insufficient volume of pus to collect via syringe (<1 mL), two wound swab samples using a sterile cotton swab (Copan Eswab kit, COPAN Diagnostics, Murrieta, CA, USA) were collected. The size of the wounds ranged from 1 cm × 1 cm to 20 cm × 15 cm. The samples were immediately stored in dry ice followed by storage at −80 °C until processed. 

### 2.2. Screening for Methicillin-Sensitive and Methicillin-Resistant S. aureus

A ten to fifty microliter sample of the pus material was streaked on the blood agar plates (BAP) and subsequently on mannitol salt agar (MSA) plates to isolate *S. aureus*. Wound swabs were streaked onto BAP followed by streaking the suspected colony on MSA plates. The suspected *S. aureus* colonies were confirmed by the Staphaurex latex Agglutination Test (Thermo Fischer, Waltham, MA, USA). Identification of MRSA was confirmed by identifying the presence of the *mecA* gene using polymerase chain reaction (PCR) as previously described [19]. Identification of the seven non-*S. aureus* isolates was done using matrix-assisted laser desorption/ionization time-of-flight mass spectrometry (MALDI-TOF MS) [25]. The *spa* typing was performed by the method described in [26] and spa types were determined by using the Ridom database (http://spaserver.ridom.de/, accessed on 1 March 2022).

### 2.3. RNA Extraction and cDNA Synthesis

Total RNA from the pus or swab samples was extracted using TRIzol reagent. The swab was transferred to a 2 mL centrifuge tube containing 500 μL of Ramel MH broth and 1 mL RNAprotect bacteria reagent. The sample was incubated at room temperature for 5 min and centrifuged at maximum speed for 5 min to pellet the cells. The supernatant was discarded and 1 mL TRIzol was added to the cell pellet. The TRIzol mixture was transferred to a 2 mL lysing matrix tube and homogenized for 20 s. Two hundred microliter of chloroform was added to the tube, and the homogenate was centrifuged at high speed for 10 min to separate into a clear aqueous upper layer containing RNA. RNA from the aqueous layer was precipitated with 500 μL of isopropanol and was purified using Qiagen’s RNeasy kit. DNase treatment was then performed using Ambion’s DNase treatment kit. RNA was converted into cDNA using the Life Technologies high-capacity cDNA reverse transcription kit.

### 2.4. cDNA Sequencing

Total cDNA samples were purified and concentrated using the QIAquick PCR purification kit. Libraries were prepared for sequencing using the KAPA HyperPlus Library Preparation Kit as described below. Depending on the quantity and quality of cDNA available for each sample, 1–50 ng of starting material was enzymatically fragmented for 2 min at 37 °C. The ends of each fragment were enzymatically repaired, with 5′ phosphorylation and 3′ A-tailing. Illumina (San Diego, CA, USA). TruSeq universal adapters (IDT, Coralville, IA, USA) were duplexed in solution by annealing together at a concentration of 3 µM and ligated to the end-repaired fragments. Ligation reaction products were cleaned up using Agencourt AMPure XP beads (Beckman Coulter, Indianapolis, IN, USA). Libraries were amplified using iTru5 and iTru7 primers, to incorporate dual eight bp indexes. PCR products were cleaned up using size selection beads. The final cDNA libraries were sized using the TapeStation (Agilent Technologies, Santa Clara, CA, USA) and quantitated by qPCR using a KAPA library quantitation kit (KAPA Biosystems, Wilmington, MA, USA). The libraries were pooled in equimolar ratios, diluted to 10 pM, and sequenced on a MiSeq instrument using the V3 150 cycle reagent kit (Illumina, San Diego, CA, USA) [27]. 

### 2.5. Data Analysis

Two bioinformatics tools, BWA (0.7.13) and Bowtie (2.2.7) were used to assemble the raw sequences as paired-end fastq files and map the output reads to well-annotated USA300FPR3757 genome to match the cDNA and identify the transcripts. The fraction and counts of reads for the coverage of the coding region of the genes (CDS regions) were processed using the SAMTOOLS v.1.3 with the default settings on the annotated reference *S. aureus* genome, USA300FPR3757. This process was then applied for each of the samples that generated a usable cDNA count. Summary tables of all the genes for all the samples were generated as per each assembler and the reference genome. Estimation of relative cDNA counts was calculated by dividing the cDNA count of genes of interest by *gmk* (guanylate kinase), a housekeeping gene [28,29] Welch’s *t*-test was used to compare the relative cDNA counts between pus and swab samples to determine the significant *p* values. A *p* value of ≤0.05 was considered significant.

## 3. Results and Discussion

**Figure 1:**
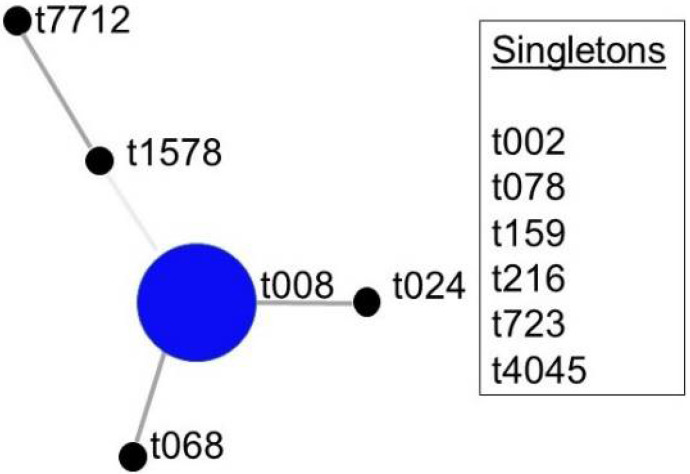
Based upon repeat pattern (BURP) analysis of the 11 *spa* types, *spa*-CC t008 was the major clonal complex and there were six singletons.

We enrolled 53 patients with SSTI in this study. The average age of the *S. aureus*-positive subjects was 42.33 years and 17 of the 30 (56.67%) were male. Thirty subjects yielded *S. aureus* on a BAP from the wound sample. All *S. aureus* isolates were further confirmed by the latex agglutination test. Seven samples that were negative for *S. aureus* grew *S. caprae*, *S. capitis*, *S. epidermidis*, *S. lugdensis*, *S. homimis*, and *Citrobacter braakii* as major bacteria confirmed by MALDI-TOF. Twenty of the *S. aureus* isolates were MRSA as determined by the presence of the *mecA* gene by PCR and 10 were MSSA. *S. aureus* isolates from twenty-eight samples were represented by 11 *spa* types, t008 = 17; t024 = 2; one each of t002, t068, t078, t159, t216, t723, t1578, t4045, t7712, and two isolates were non-typeable (Figure 1). Eight SSTIs were large enough to yield a volume of purulent material while the remainder (*n* = 20) of the samples were collected by a sterile swab from the surface of the wound. cDNAs could be made only from 23 *S. aureus* positive samples. Six specimens out of 23 *S. aureus* positive samples yielded less than 50 total *S. aureus* cDNA counts and hence they were excluded from further analysis as were the non-*S. aureus* samples. 

All cDNA samples yielded sequences that had *S. aureus* gene transcripts. Total sequencing reads per sample ranged from 3,867,530–7,552,309 (average: 5,432,957) (Table 1). Of these cDNA reads, 43,660 on an average matched with a locus on a *S. aureus* USA300 genome (Table 1). Expression of as many as 858 *S. aureus* genes was observed of which 293 genes had at least five cDNA reads. The relative cDNA counts of the genes of interest were measured against the cDNA counts of *gmk* because of its relatively constant expression during growth phases [28,29].

The virulence factor genes that were prevalent at the site of infection were mainly PVL genes, *lukSF-PV,* and hemolysin genes, *hla*, *hld*, *hlgB*, and *hlgC* (Table 2). PVL has a high cytolytic activity to polymorphonuclear leukocytes and macrophages [30,31,32,33,34]. Subunits LukS-PV and LukF-PV assemble and oligomerize on host cells to form β-barrel transmembrane channels that cause osmotic dysregulation and eventually lead to necrosis [35,36,37,38]. These cytotoxic effects are aggravated when they combine with other toxins such as gamma hemolysin [35,37,39].

Our data showed that PVL is perhaps the most significant toxin produced in SSTI as seen from both the pus and swab samples. Since PVL usually correlates with the severity of skin infections [40], therefore, smaller wounds whose samples could only be collected using a swab showed a lower expression of PVL. Interestingly, Lina et al. (1999) reported that PVL was only expressed in necrotic wounds and was completely absent from superficial folliculitis [41]. Indeed, in a transcriptome study, both PVL and gamma hemolysin expression were found to be several-fold higher in human cutaneous abscess samples (*n* = 3) when compared to the cultured USA300 strain and mouse infected kidneys in a non-SSTI model [42]. PVL positive strains are often associated with severe persistent and recurrent SSTI [6,10,34,43,44]. Previous studies measuring the transcript levels of virulent genes such as PVL, *hlgB*, *hla*, and *lukE* in cutaneous infections have observed several-fold higher expression levels in them, compared to the same isolates grown in vitro [42,45]. Secretion of these toxins to the cell exterior passing the highly cross-linked peptidoglycan requires enzymes such as IsaA that cleave peptidoglycan. Immunodominant staphylococcal antigen, *isaA* that was expressed at the site of infection (Table 2) is a lytic transglycosylase, which cleaves peptidoglycan [46]. The immunodominant staphylococcal antigen is highly immunogenic and produces high antibody titers in individuals with *S. aureus* infection [47]. Treatment with monoclonal antibodies against IsaA protected mice from bacteremia, however, this protection was not explored in SSTI models [48]. Interestingly, most of the virulence genes showed a significantly higher expression in the pus samples (Table 2, Table 3 and Table 4) than in the swab samples. However, *hlgB*, *hld*, and *hla* were high in both pus and swab samples with no special preference to any of these sample types. 

Transcripts for virulence genes that were not detected in this study were *seb*, *sec*, *sed*, *see*, *seh*, *sej*, and *seg*2, suggesting their lack of role in SSTI. Although a previous study had detected the presence of enterotoxin gene *seb* to be more prevalent in *S. aureus* isolated from SSTI samples and *sea* to be predominant in *S. aureus* isolated from non-SSTI samples, their expression levels remain indefinable [49]. Transcripts of seven housekeeping genes of the multilocus sequence typing scheme were also identified, although their cDNA count averages (1.2 to 7) were low. 

Adhesion and clumping factor genes that were detected at a higher level were for autolysin, clumping factors B and laminin-binding protein. (Table 3). Autolysin [50,51,52] and clumping factors [53,54,55] are the early proteins involved in adhesion and biofilm formation. Autolysins are the peptidoglycan hydrolases that are suggested to contribute to the appearance of perforating holes throughout the cell wall [56] that may help in the secretion of toxins that would otherwise be contained due to the constraints imposed by the three-dimensional mesh of the cell wall. All of the adhesion and clumping transcripts showed a significant expression in the pus than the swab samples.

Both ClfA and ClfB strongly bind to plasma fibrinogen and cytokeratin promoting bacterial colonization and biofilm formation at the site of a wound during SSTI [57,58,59]. Transcripts for adhesion genes such as *eno* (laminin-binding protein), *fnbA* and *fnbB* (fibronectin-binding proteins A and B), *coa* (coagulase), and *ebps* (elastin-binding protein) were also detected (Table 3). Apart from adhesion, they are also involved in blood clumping, platelet activation, and the internalization of *S. aureus* into host cells [60,61,62]. Surprisingly, we did not detect coagulase transcripts in the swab samples. We also observed transcripts for regulatory genes, accessory gene regulators, *agrC* and *agrB*, and *S. aureus* exoprotein, *saeS*, and *saeR* from the purulent samples (Table 4). They all showed significant differences between the pus and the swab samples except for *agrB*.

Mutations in *agr* in *S. aureus* in murine skin infection models have been linked to smaller lesion sizes due to less hemolysin expression and low bacterial burden compared to the wild-type strains [63,64]. *Agr* (RNAIII) upregulates *hla*, *hlg*, *hld*, *lukAB*, *lukGH*, and downregulates cell wall secretary protein IsaA [65,66]. SaeRS is a two-component regulatory system that upregulates *coa*, *hla*, *hlb*, *hlg*, PVL, and *isaA* [67,68,69,70]. Both *atl* and *isaA* were found to be upregulated in tissues isolated from USA300-positive cutaneous abscess samples [42]. The *atl* is a major autolysin and plays an important role in cell division and biofilm formation [52,71]. In this study, staphylococcal superantigen such as (*ssl*) and putative virulence genes such as lipoprotein lipases *(lpl*10) genes were identified infrequently.

The complexity of the *S. aureus* pathogenesis is due to the production of different virulence and adhesion proteins at different stages of the disease process. Its microbial surface components recognizing adhesive matrix molecules (MSCRAMMs) help in binding to the host’s extracellular matrix and sometimes more than one molecule bind to the same tissue. Expression of these molecules usually occurs in the colonization phase in contrast to the secreted toxins in the late infection phase [72] In this study, we observed significant differences in gene expression of toxin and adhesion genes between the pus and the swab samples. The higher expression of these genes could be either due to (i) higher density of the *S. aureus* in the pus, and/or (ii) due to influx of immune cells at the wound site causing higher levels of inflammation. Indeed, in a mouse model study, a higher inoculum of S. aureus increased the inoculum size [73] and direct challenge with purified PVL in a rabbit pneumonia model caused lung damage by cytotoxic granules from the recruited polymorphonuclear at the site of the challenge [74]. 

It is important to note some limitations of this study. While we detected several important genes for virulence proteins such as PVL, hemolysin, and autolysin, the average cDNA counts of putative genes such as *lpl*10, *seq*, *sek*, etc., were below the average cDNA cut-off except for the *ear* gene. The overall low cDNA counts matching the *S. aureus* were likely due to (1) most abscesses contained insufficient purulent material and required sample collection via wound swab, which reduced the amount of *S. aureus* available for transcript analysis, and (2) a limited number of *S. aureus* cells present in the wound swab or even the purulent material. These limitations can be controlled by using larger volumes of purulent material to measure transcript levels. This study establishes a foundation for future studies involving larger cohorts with larger volumes of purulent material. In conclusion, interrogation of in situ *S. aureus* gene expression in SSTI identified a number of known toxin and adhesion/clumping factor genes, suggesting that *S. aureus* SSTI pathogenesis is likely due to a variety of virulence proteins acting in an orchestrated manner.

## Figures and Tables

**Table 1:** Number of cDNA reads at different steps of data processing.

cDNA Reads	Minimum	Maximum	Average
Range of sequencing reads per sample	3,867,530	7,552,309	5,432,957
Ranges of cDNA reads that matched to *S. aureus* USA300	255	247,463	43,660
Ranges of number of sequences that match to an annotated loci	0	77,765	11,345
Ranges of the number of loci that have at least one sequence of coverage	0	2372	668

**Table 2:** The average cDNA count of the virulence genes from pus or swab samples and their relative cDNA count to that of *gmk*.

Virulence Genes	Gene	Pus (*n* = 7)		Swab (*n* = 10)		*p* Value **
		Average cDNA Count	cDNA Count Relative to *gmk* *	Average cDNA Count	cDNA Count Relative to *gmk* *	
Panton–Valentine Leukocidin	*lukF-PV*	235.4	22.6	6.8	11.3	0.06
Panton–Valentine Leukocidin	*lukS-PV*	194.4	18.6	8.5	14.2	≤0.05
Immunodominant staphylococcal antigen	*isaA*	139.3	13.4	5.1	8.5	≤0.05
Staphylococcal SecretoryAntigen	*ssaA*	49.9	4.8	1.9	3.1	≤0.05
Hemolysin D	*hld*	39.3	3.8	2.8	4.6	0.06
Hemolysin B	*hlgB*	26.6	2.6	0.4	0.7	0.10
Hemolysin C	*hlgC*	15	1.4	0.8	1.33	≤0.05
Hemolysin A	*hla*	0.7	1.3	0.8	1.33	0.09

* The average cDNA count of *gmk* were 10.43 and 0.6 in pus and swab samples, respectively. ** *p* values are for comparison between pus and swab samples.

**Table 3:** The average cDNA count of the adhesion and clumping factor genes from pus or swab samples and their relative cDNA count to that of *gmk*.

Adhesion and Clumping Factor Genes	Gene	Pus (*n* = 7)		Swab (*n* = 10)		*p* Values **
		Average cDNA Count	cDNA Count Relative to *gmk* *	Average cDNA Count	cDNA Count Relative to *gmk* *	
Autolysin	*atl*	185.4	17.7	5	8.33	≤0.05
Clumping factor	*clfB*	110.3	10.57	7	11.6	≤0.05
Laminin-binding protein	*eno*	63	6.04	1.9	3.16	≤0.05
Fibronectin-binding protein	*fnbA*	61.9	5.93	0.2	0.33	≤0.05
Clumping factor	*clfA*	40.9	3.9	0.5	0.83	≤0.05
Fibronectin-binding protein	*fnbB*	39.1	3.75	0.4	0.66	≤0.05
Coagulase	*coa*	17.3	1.65	0	0	≤0.05
Elastin-binding protein	*ebpS*	13.6	1.3	0.7	1.16	≤0.05

* The average cDNA count of *gmk* are 10.43 and 0.6 in pus and swab samples, respectively. ** *p* values are for comparison between pus and swab samples.

**Table 4:** The average cDNA count of the regulatory genes from pus or swab samples and their relative cDNA count to that of *gmk*.

Regulatory genes	Gene	Pus (*n* = 7)		Swab (*n* = 10)		*p* Value **
		Average cDNA Count	cDNA Count Relative to *gmk* *	Average cDNA Count	cDNA Count Relative to *gmk* *	
*S. aureus* exoprotein expression S	*saeS*	66.7	6.3	0.8	1.33	≤0.05
Accessory gene regulator C	*agrC*	58.6	5.6	0.9	1.5	≤0.05
*S. aureus* exoprotein expression R	*saeR*	56.3	5.4	2	3.33	≤0.05
Accessory gene regulator B	*agrB*	25.6	2.45	0.5	0.83	0.073

* The average cDNA count of *gmk* were 10.43 and 0.6 in pus and swab samples, respectively. ** *p* values are for comparison between pus and swab samples.

## Data Availability

All the data is available on request to the corresponding author.
